# Anna Livia Plurabelle’s normless interactions in *Finnegans Wake*

**DOI:** 10.3389/fpsyg.2022.917865

**Published:** 2022-10-06

**Authors:** Hamid Farahmandian, Zhang Jian-qing

**Affiliations:** ^1^School of Foreign Languages, Chengdu University, Chengdu, China; ^2^School of Foreign Languages, Zhejiang Ocean University, Zhoushan, China

**Keywords:** James Joyce, *Finnegans Wake*, ALP, neurotics, Karen Horney

## Abstract

The complex structure and characterization of James Joyce’s *Finnegans Wake* present a normless environment for its female characters, specifically Anna Livia Plurabelle (ALP). This paper explores ALP’s normlessness in her social interactions using Karen Horney’s theory of neurosis as a methodological device to reveal ALP’s detached personality. Horney considers normlessness a trend of “Moving Away from People,” also known as a detached personality. According to Horney, “self-sufficiency,” “perfection,” and “narrow limits in life” are the three neurotic factors that produce a detached personality, all of which are apparent in ALP’s personality. In this novel, ALP is portrayed as neurotic to demonstrate her dependence on men and how she coopts male power in favor of her needs.

## Introduction

Norms are behavioral standards and obligational states that individuals must adhere to. They are expectations of an individual’s behaviors, which take “the form of a rule that is socially rather than formally enforced” (Durkheim, 1984). According to Shah, “many of the problems of personality as well as society are mostly the problems of non-conformity to norms” (Shah, 2017). In contrast, normlessness represents a condition where “the social norms regulating individual conduct have broken down or are no longer effective as rules for behavior” (Seeman, 1993). Thus, as an individual sheds social norms, he or she experiences alienation, isolation, and estrangement both in public and private settings. In other words, an individual, bearing a normless personality, “stubbornly rejects all conventional rules and standards” (Horney, 2013). Familiar with psychoanalysis theories, Joyce (1882–1941) presents his later works, *Ulysses* (1922) and *Finnegans Wake* (1939) not pervert but more neurotic in style. The theory of neurosis, later on, was elucidated and explained by Horney (1885–1952).

Horney, in her theory of neurosis, describes this situation as a trend of “moving away from people,” also known as a detached personality. According to Horney, people with detached personalities must not love, hate, cooperate with others, or become involved in any way. Their driving principle is never to become so attached to people or objects that they become indispensable, as “[t]hat would jeopardize aloofness; better to have nothing matter much” (Horney, 2013). Unlike Sigmund Freud, who insists on the innate biological differences in the personalities of women and men, Horney associates these differences with society and culture of a specific time and place rather than biology.

In Joyce’s *Finnegans Wake*, Anna Livia Plurabelle (ALP), the wife of Humphrey Chimpden Earwicker (HCE), is the river-woman, who opens the novel and her monolog concludes the book and frequently transforms throughout the novel in different forms of “mythic, natural, cultural, and historical human manifestations across time and space, even as she is unbound by time or space” (Reilly, 2020). In response to the contextualization of the Irish social and ideological context with the characteristic of female and male power relations, which Joyce problematizes in his works and “an unbridled and threatening female sexuality” (Lovejoy, 2017) in the 1920s and 1930s Ireland, in this paper, I suggest that ALP is portrayed as a neurotic woman–not pervert, which is commonly known–who tries to accomplish her needs by adopting a normless personality. Based on Horney’s definition of neurosis, I will study how ALP’s detached personality compels her to move away from people and makes her neurotically alienated because of the following three needs she struggle for in society. For the societal position of women and the cultural position of men, featuring with power and prestige, in back-then Ireland, Joyce portrays ALP as a woman, who is in serious need for “superiority,” “extreme self-sufficiency,” and “narrow limits on her life.” In other words, because of psychological conflict, ALP tries to avoid society to remain comfortable by dependence on men and the way she coopts male power in favor of her needs and wishes. While several researches have been done on psychological study of Joyce (Norris, 1974; Thurston, 2004; Lacan, 2016; Bristow, 2017; Henke, 2017; Adams, 2018), they overlook the specific study of feminine personality from the perspective of neurosis in *Finnegans Wake*, which I hope to present in this paper.

## Anna Livia Plurabelle’s sense of superiority

A person with a sense of superiority sees herself as more valuable or talented than others, a trait that makes an individual apathetic and isolated. In the novel, ALP overlooks other women and distances herself from them: “I’m loothing them that’s here and all I lothe” (Joyce, 2012). She feels that this behavior will make her perfect and set her apart from others even if it leaves her “Lonely in […] loneness” (Joyce, 2012). This is in an obvious parallel with Horney’s idea of Neurosis, where the person involved is “provoked by the gap between the idealized and perfect image of the self and the less-than-perfect reality” (Farahmandian and Shao, 2022). She purposefully alienates herself to avoid the anxieties that accompany living in society. Her lack of social interactions combined with a feeling of superiority and inflated sense of heterosexuality inspire her neurotic need for perfection. Therefore, ALP’s sense of superiority is revealed in three aspects, including her dependence on male power to demonstrate her excellence, inflated sense of heterosexuality, and the sense of self-confidence.

A sense of superiority is primarily caused by a strong dependence on power and external sources while excessive practice of this dependence may bring about normlessness. One aspect of sense of superiority in “Annah the Allmaziful, the Everliving” (Joyce, 2012) is her use of masculine power to enhance her social position. According to Miller (1996), “While Joyce implies the deity may never rise, the women of *Finnegans Wake* are portrayed as consistently focused upon male power.” Evidence of ALP’s need for masculine power to fuel her sense of superiority is found in the letters she writes. Claudine Raynaud believes that the letters of *Finnegans Wake* are inescapably centered upon masculinity: “he would pen for her, he would pine for her” (Joyce, 2012).

The letters are a product of male desires, fears, and guilts: the writing master Shem/Jerry makes Issy write them. Bypassing her, or rather through her, since a woman is man’s mirror, the penman writes letters to himself emulating Swift and his correspondence with Stella (Raynaud 315).

In this case, a woman serves as the conduit for the male creator and the practice of teaching letter writing to women serves only to allow men to recreate themselves: “man’s enterprise to teach a woman how to write his desire is also an acknowledgment of her exile from language” (Raynaud, 1986). Thus, Joyce illustrates women’s alienation from their creativity and consciousness, which indicates masculine social domination. Reflecting a traditional attribute of many cultures, “the Wakean religion of HCE’s second coming is also the source of ALP’s desire for male children, and to this effect, he” “cleared out three hundred and sixty five idles to set up one all khalassal for henwives hoping to have males” (Joyce, 2012). Consequently, ALP’s intention to use male power in favor of her own needs and to demonstrate her superiority over other women seems rather neurotic.

The inflated sense of heterosexuality becomes another justification for ALP’s sense of superiority. Heterosexuality refers to sexual behaviors and sexual or romantic attraction between opposite-sex individuals. While it is the most common sexual orientation, “a higher and uncontrollable” (Jeffrey and Barata, 2019) degree of heterosexuality is normless and neurotic, affecting individuals’ personalities and social bonds. In *Finnegans Wake*, ALP feels superior to other women and tries to distance herself from them. Her sexual conducts present her as the “waters of babalong” (Joyce, 2012) and later a “scarlet thread” (Joyce, 2012). As red as blood, which is in circulation constantly, ALP’s hair signifies the stimulating female sensuality that her husband is incapable of satisfying and the blood, which keeps generating families.

Anna Livia Plurabelle wants sexual attention from any man who can boast of her superiority, even in the absence of her husband: “A coneywink after the bunting fell. Letting on she didn’t care, sina feza, me absentee, him man in possession, the proxenete!” (Joyce, 2012). This detachment from femininity forces ALP into exile and self-alienation. ALP claims that she would happily exchange her newfound voice for the silence of HCE’s tomb, saying, “I whisht I wast be that dumb tyke and he’d wish it was me yonder heel” (Joyce, 2012). She cannot imagine life without the husband’s power; therefore, she wishes to substitute him immediately after his death. Should she lose her male support, she would be unable to assert her superiority over other women any longer. According to Miller (1996), “it is perhaps female desire for the male at the end of time which restarts the cycle of the *Finnegans Wake*, just as masculine desire results in the biological second coming.” Furthermore, Margaret Solomon takes a symbolic view of ALP’s speech, suggesting that the letter “T” in “the,” ALP’s closing word in the novel like “tree,” “tea,” and “three,” Solomon (1969) is phallic and represents a new cycle of sexual desires over the deceased HCE.

Thus, Joyce wants to show the normality of life’s continuation through the normlessness of ALP’s need for heterosexuality. Similar to Molly’s belief in the deity in the Penelope episode of *Ulysses* (1922), ALP overtly associates herself with an infinite and omnipotent masculine deity, in which the incessant demand for heterosexuality proves her normlessness and neurotic personality. Since she may become anxious if she cannot satisfy her desire for a man to support her, she needs to replace her husband, whose death is imminent, with another man.

Furthermore, a sense of superiority also leads to crashed self-confidence. Self-confidence denotes reliance on one’s “abilities, qualities, and judgment” (Edison, 2016). However, too much self-confidence can lead to alienation in social interactions. For instance, due to her over-abundant self-confidence, ALP believes that she is the savior of her husband. Thus, when her husband, HCE, is accused of a sexual crime in Phoenix Park, “the murky incident that serves as the Wake’s original sin” (Kaufman, 2020), she attempts to exonerate him with a letter, “untitled mamafesta” (Joyce, 2012), which is unusual. The letter is composed by ALP, written by Shem the Penman, and delivered by Shaun the Postman. However, the letter is not delivered to its destination. Instead, Biddy, the hen, intercepts the letter, which is supposed to vindicate HCE. ALP’s letter appears in different forms several times throughout the novel, and, because its actual content is not described, critics (Burgess, 1967; Herring, 1987; Henkes and Bindervoet, 2004) generally suppose it is both an accusation and exoneration of HCE’s sin. Book IV of *Finnegans Wake* includes the complete text of a letter ALP writes to the world about her husband’s innocence. However, Philip Herring believes that “[t]he effect of ALP’s letter is precisely the opposite of her intent […] the more ALP defends her husband in her letter, the more scandal attaches to him” (196). Patrick McCarthy also claims that “[i]t is appropriate that the waters of the Liffey, representing Anna Livia, are washing away the evidence of Earwicker’s sins, […] for she takes on her husband’s guilt and redeems him; alternately she is tainted with his crimes and regarded as an accomplice” (McCarthy, 2005). ALP anxiously wishes to clear HCE of his crime because, if she fails, she will no longer be able to declare her power and superiority over other women. Hence, ALP struggles both publicly and privately to exonerate her husband, though she fails in the end. Of course, ALP does not proclaim his innocence out of love but to serve her own needs, using HCE as a means to attain her own status in society. In summation, ALP’s lack of love for her husband is demonstrated by her strong heterosexual desire, overstated self-confidence, and sense of superiority over other women.

Anna Livia Plurabelle’s overstated self-confidence gives her a sense of superiority and a detached personality, which is also influenced by her dependence on male power and normless heterosexuality. ALP fails in the three aforementioned aspects of her sense of superiority and thus becomes alienated from and frustrated by society. This sense of frustration only makes her more neurotic and detached from others.

## Anna Livia Plurabelle’s frustrated claim for self-reliance

Self-reliance is depending on one’s own resources and power rather than those of others. Normless self-reliance causes frustration and alienation from others. ALP shows that, to attain perfection, she must rely solely on her own abilities, leading her to disconnect from any outside resources. Joyce attributes non-normative features and duties to ALP that cause her to become detached from others. Thus, as being discussed below, in order to present her self-reliance, ALP tries to be charitable toward her children, be independent of her husband, and be away from any changes in life.

Failed maternal charity is the first result of ALP’s frustrated attempts at self-reliance. Maternal charity denotes a mother’s kindness while raising her children. ALP, beyond child-raising and childbearing, experiences a radical change in the mix of self-reliance and charity, a phenomenon that occurs in many retired couples, according to John Anderson, who understands this change using “the Vico Systemization of opposites where the opposites fluctuate from pole to pole as one goes up while the other goes down but with a tendency to reconcile; independence and charity are opposites of the extent that compulsion of charity, which inevitably involves doing what you want” (17).

Now changed, ALP reconciles these opposites by maximizing both, which is “a real hat trick, especially in connection with death” (Anderson, 2014). As an example, in chapter 3.4, ALP treats Shem to a wet-bedside lesson where she provides her son with an act of maternal charity. However, in doing so, ALP neglects her husband’s desire “to keep humping his poke” (Anderson, 2014). Nevertheless, afterward, she returns to HCE with extra favor to limit his choices by keeping him reliant on the family’s support. In these efforts, ALP is charitable, and she only passes self-reliance from the father to the son. Unfortunately, ALP’s attempt at self-reliance fails, and the novel implies that absent child-raising in that condition is the ultimate charity. In other words, ALP “leaves off controlling her children and husband,” (Anderson, 2014) and tries to lessen the burden of life’s responsibilities. Ultimately, at the time of HCE’s death, ALP is unsuccessful at balancing self-reliance with charity. As a result, it becomes apparent that she cannot take on her husband’s role in raising the children; likewise, she cannot take on the role of the children for their father. Therefore, without her self-reliance, she becomes neurotic about what she may lose.

Regretting life’s typical changes is the second result of failed attempts at self-reliance. Such regret leads to normlessness and alienation. Joyce portrays ALP as a universal character representing motherhood, wifehood, and femininity. ALP is strong enough to defy norms despite the resulting failures. Although ALP wishes to appear strong to cope with her past and future responsibilities and problems, she fails to achieve this strength. ALP is unable to depend on her own strength during normal life because she cannot cope with life’s changes. Insisting on the stability of life, she takes her husband on a journey of her body from past to present. Although they romantically march across her body’s landscape during lovemaking, even ALP’s “littleeasechapel” (Joyce, 2012) is a water-closet, which is not only romantic but also changeable. As she tries to ignore the ongoing life’s changes, she continues struggling, saying, “I will tell you all sorts of makeup things, strangerous; and show you every simple storyplace we pass” (Joyce, 2012). ALP takes her husband on this journey to show that although the “places […] are made up, […] every feature of her woman’s body is constructed by social codes so that her villages and valleys are vile vanities, and they are veiled with language” (Brivic, 1995), as they emerge merely as a series of signs. Hence, the text returns to her childhood to show how she became so commodified. The tour of the city proceeds as a desperate and isolated version of past tours, and ALP notices the changes and feels weak. She feels the wind pierce her and reaches a crucial point, saying, “Here, weir, reach, island, bridge” (Joyce, 2012). McHugh notes that the Liffey is tidal to Island Bridge, so this is where the river meets the sea: “where you meet I” (Joyce, 2012). It is implied that the “you” she addresses is in connection with “the father ocean she enters at the end, that husband and father are confused for her” (Brivic, 1995). Joyce reveals that an individual who wishes to rely on her abilities and to move away from others should have ample resources and be strong enough to cope with the reactions to her alienation. Joyce’s ALP is characterized as a person who should depend on her strength, knowledge, and abilities to obtain her needs and desires; however, she fails in the end. ALP cannot tolerate the normal changes in life and becomes anxious about her lack of abilities when she cannot control them. This failed self-reliance makes ALP neurotic. As a result, her detached personality, which is greatly dependent on her ability to meet her own needs, is defeated.

Joyce advances lost salvation as the third and final outcome of a frustrated attempt at self-reliance. Salvation denotes a means, cause, or source of being protected or saved from harm. In religious terms, it also entails the restoration or ascension of the natural world to a higher realm or state. Joyce demonstrates that, if using self-reliance to achieve salvation is doomed by supernatural elements, an individual can easily become normless relative to her religion and society. In *Finnegans Wake*, ALP moves away from humanity to attain salvation. At the beginning of the novel, it appears that ALP, as a “riverrun” woman, is capable of reaching the ocean as her final salvation. Therefore, we acknowledge her reason for feeling superior to others and accept her reliance on her own strength to maintain this superiority. However, as the novel progresses, ALP’s powerlessness to fulfill this wish becomes apparent. Joyce shows us that ALP “at the moment of reaching her epiphany-like self-realization in individual and non-fearful identity, even throwing off the Eden sponsored fear of death” (Anderson, 2014). This is a new experience for her and would seem to constitute her liberation as she, as a river, ascends to merge into the god. However, the final page of *Finnegans Wake* depicts her being immediately reincarnated and going down. Her inability to achieve salvation is the central theme of this chapter. Since she will not be liberated, ALP does not make it to the unbounded ocean, which represents liberation, but only makes it to Dublin Bay. As suggested by the local cleansing gulls, “the Bay represents her cleansing and rebirth vehicle for reincarnation, (i.e.) the amniotic waters of her reincarnation mother” (Anderson, 2014).

As a result, ALP is defeated because of her normless trust in her own power to reach salvation and ultimate freedom. Joyce represents this failure through ALP’s fall into a bay instead of an ocean. ALP is neurotically detached from humanity as she cannot accomplish the duties that her society assigns her. However, ALP insists on her failure since a neurotic person refuses to recognize his or her real self and abilities. Therefore, she struggles and ultimately fails to take on her husband’s duties in raising her children. In addition, ALP fails to concede to life’s normal changes and to realize her inability to attain salvation through her own power, condemning her to reincarnation instead. These three elements pave the way for the failure of her over-stated self-reliance. The resulting collapse demonstrates ALP’s detached, neurotic personality and alienation.

## Anna Livia Plurabelle’s desire for narrow limits

Placing narrow limits on one’s life might allow an individual to feel less responsible for herself and other people. In *Finnegans Wake*, ALP’s detached personality restricts her duties and responsibilities to relieve the pressure that affects her detachedness. If ALP wishes to alienate herself to lead a solitary life, she must limit the anxieties caused by other people. For example, her indifference to serious issues such as her husband’s sexual crime proves her inability to deal with her frustration. Likewise, her avoidance of any violence or conflict limits her life. Finally, by prioritizing herself or her daughter on any issue, ALP limits her life by distancing herself from her other family members. Thus, ALP narrowly limits her life to ease her anxiety and attain peace.

Indifference to marital normlessness arises initially from the neurotic need to limit one’s life and is represented as “a diminished quality of life” (Widiger and Oltmanns, 2017). Marital life is based upon predictable norms, and indifference to those norms makes an individual normless. Normlessness in marital life originates from the feeling of distancing oneself from its responsibilities. Unlike Bloom in *Ulysses*, who appears incapable of dealing with his wife’s infidelity, Alp becomes indifferent to her husband’s sexual scandals. When ALP understands that “he is not what he used to be” (McCarthy, 2021), ALP does not complain about HCE’s scandal; she even seems to enjoy his behavior. ALP tries to rouse HCE to a productive state by various ways like cooking: “she’d cook him up blooms of fisk and lay to his hearts foot her meddery eygs” (Joyce, 2012), erotic encouragements: “throwing all the neiss little whors in the world at him!” (Joyce, 2012), and letter-writing letter: “For the putty affair I have is wore out, so it is, sitting, yaping and waiting for my old Dane hodder dodderer […] to wake himself out of his winter’s doze and bore me down like he used to” (Joyce, 2012).

In terms of sexual demands, ALP might understand just how “polymorphously perverse he is and how, if it is to be revived, his sexuality must be addressed at a level beneath differentiation” (Beckman, 1995). As a stagnant man, HCE demands that ALP satisfies his needs in multiple ways and relies on her constant activities and attention for motivation. ALP gives life as she destroys, making her one of the main sources of frustration in HCE’s dream. Hitherto, he cannot rid her from his thoughts because she is both the source of, and the only potential cure for, his impotence. Moreover, in defending HCE for his sexual crime at the park, ALP seems pleased about her husband’s impropriety because she admits that she cannot satisfy her husband’s desires. However, ALP still needs HCE to protect her with his masculine power. Therefore, she cannot show disapproval if her husband is sexually deviant. Besides, ALP is apathetic to HCE’s crime to avoid potential arguments or violence. Thus, it is possible that ALP is happy to have her husband relieve her of responsibility for his sexual desire, although this indifference makes ALP normless in her marital life and detached from her social life.

Moreover, the need to wash away life’s challenges arises from the desire to limit one’s life. To remove dirt, one must wash it away. Throughout the first seven chapters of *Finnegans Wake*, Joyce represents ALP as someone who washes away controversy and conflict because she cannot tolerate the anxiety that accompanies social or family conflicts. In addition to ALP’s indifference toward her husband’s crime, she is also apathetic and helpless to intervene in the conflicts of her children, Shem and Shaun. ALP, like a river, flows between her children as if they are riverbanks and cannot bring “their opposition into unity, nor does she erase their differences. Rather, the difference is placed under erasure: it is scored through in to present condition only to underscore, to highlight and embrace, another logic of dissimilarity” (Eide, 2002). Therefore, she refuses to interfere in their violence to avoid anxiety. In brief, in *Finnegans Wake*, Joyce argues that avoidance of social conflict may result in an individual’s desire to distance herself from anything that provokes anxiety.

Prioritizing certain behaviors in one’s children also demonstrates the neurotic need for limited space to reduce the responsibility of sharing affection. Despite her rejection of femininity, ALP favors her daughter, Issy, over her sons. Issy is like a young ALP “whose showers feed the streams that form the Liffey and who is more knowing than her young years” (Cliett, 2012). ALP focuses more affection on her daughter than her sons. Although ALP needs male power for protection and to help her accomplish her desires, she privileges femininity over masculinity with regard to the future. For example, ALP wants her daughter to inherit the house keys, which are symbolic of the future generation: “We pass through grass behosh the bush to. Whish! A gull. Gulls. Far calls. Coming, far! End here. Us then. Finn, again! Take. Bussoftlhee, mememormee! Till thousendsthee. Lps. The keys to. Given! A way a lone a last a loved along the” (Joyce, 2012). In this unfinished sentence, which joins with the “riverrun” at the start of Finnegans Wake, ALP “passes the keys from her ‘Lps’, the labial estuary of the Liffey as it blends with the sea by Howth Head; Mundanely, this gesture may be ALP-as-mother passing the house keys to Issy-as-daughter in a vision of female succession” (Eide, 2002). Thus, ALP trusts only herself or her daughter to produce future generations.

In conclusion, Joyce creates a female character, who is neurotic and normless, rather than perverse. Drawn upon Horney’s theory of neurosis, we can conclude that the neurotic ALP is the product of the cultural and societal elements of masculinity, power, and prestige, which played significant roles in the early twentieth-century Ireland. In order to find peace, ALP, relying heavily on male power, attempts to demonstrate her superiority and self-sufficiency and give limits to her life to distance herself from others; however, these issues only increase ALP’s anxiety and psychological conflict. This conflict prevents her from satisfying her needs, as fulfilling those needs requires her to avoid social bonds. This approach-avoidance conflict increases her alienation and normlessness in both public and private life.

## Data availability statement

The original contributions presented in this study are included in the article/supplementary material, further inquiries can be directed to the corresponding author/s.

## Author contributions

All authors listed have made a substantial, direct, and intellectual contribution to the work and approved it for publication.
